# Prevalence and functional impact of social (pragmatic) communication disorders

**DOI:** 10.1111/jcpp.13705

**Published:** 2022-09-16

**Authors:** Jo Saul, Sarah Griffiths, Courtenay Frazier Norbury

**Affiliations:** ^1^ University College London London UK; ^2^ Department of Special Needs Education University of Oslo Oslo Norway

**Keywords:** Social‐communication disorder, pragmatics, language

## Abstract

**Background:**

The aim of this study was to evaluate the Children's Communication Checklist‐2 (CCC‐2) for measuring social‐pragmatic communication deficits and to ascertain their prevalence and functional impact in a community sample.

**Methods:**

We used parent and teacher responses to the CCC‐2 to approximate inclusion (poor social‐pragmatic skills) and exclusion (poor structural language skills or autistic symptomatology) criteria for social (pragmatic) communication disorder (SPCD). We tested the prevalence of social‐pragmatic deficits in a population‐based sample of children (*n* = 386) aged 5–6 years old using CCC‐2 algorithms. We also investigated the academic and behavioural profiles of children with broadly defined limitations in social‐pragmatic competence on the CCC‐2.

**Results:**

Regardless of the diagnostic algorithm used, the resulting prevalence rates for social‐pragmatic deficits indicated that very few children had isolated social‐communication difficulties (0–1.3%). However, a larger proportion of children (range: 6.1–10.5%) had social‐pragmatic skills outside the expected range alongside structural language difficulties and/or autism spectrum symptoms, and this profile was associated with a range of adverse academic and behavioural outcomes.

**Conclusions:**

A considerable proportion of children in the early years of primary school has social‐pragmatic deficits that interfere with behaviour and scholastic activity; however, these rarely occur in isolation. Exclusionary criteria that include structural language may lead to underidentification of individuals with social‐pragmatic deficits that may benefit from tailored support and intervention.

## Introduction

The ability to engage in meaningful social‐communicative exchanges is a key requirement of our society, leaving individuals who struggle to use language in social contexts at greater risk for poor behavioural outcomes (Mok, Pickles, Durkin, & Conti‐Ramsden, 2014). The introduction of social (pragmatic) communication disorder (SPCD) to the Diagnostic and Statistical Manual (DSM‐5; American Psychiatric Association, 2013) provided an opportunity to formalise clinical observations and a mechanism for affected individuals to receive clinical and educational support. Nevertheless, DSM‐5 inclusion and exclusion criteria have generated considerable clinical and theoretical debate about the validity of this diagnosis and the extent to which language may contribute to social‐pragmatic deficits (cf. Flax et al., 2019; Mandy, Wang, Lee, & Skuse, 2017; Norbury, 2014; Swineford, Thurm, Baird, Wetherby, & Swedo, 2014).

SPCD is defined in DSM‐5 by four core features that include using language for social purposes (such as greetings), adapting language to contextual or listener needs, following discourse rules (turn‐taking and maintaining conversational topic) and going beyond the explicit utterance to derive intended meaning (inferencing and use of figurative language). More controversially, diagnosis requires that this symptom profile occurs in the absence of restricted and repetitive interests and behaviours associated with autism, clinically significant structural language deficits (i.e. limitations in vocabulary or grammar), or intellectual disability (APA, 2013). Applying these criteria in clinical (or research) settings has been hampered by a dearth of well‐validated, reliable and accurate diagnostic instruments (Adams, Gaile, Lockton, & Freed, 2015; Brukner‐Wertman, Laor, & Golan, 2016; Timler & Covey, 2021), and poor agreement amongst clinicians on how existing assessments align with core DSM‐5 criteria (Yuan & Dollaghan, 2018). Assessing social‐pragmatic skill in an objective way is challenging as the structure imposed by standardised tests does not represent the subtle, dynamic and complex signals that characterise natural communication, yielding a less ecologically valid evaluation (Adams, 2002).

In this paper, we adopt a broader definition of social‐pragmatic deficits (cf. SPCD) and investigate the extent to which children may have selective social‐pragmatic deficits in the absence of language impairments and/or autistic symptoms using the Children's Communication Checklist‐2 (Bishop, 2003a). The CCC‐2 was designed to ‘identify pragmatic impairments in children with communication problems’, ‘screen for children who are likely to have a [structural] language impairment’ and ‘assist in identifying children who may merit further assessment for autism’. It is therefore the only instrument currently available that assesses inclusion and exclusion domains relevant for SPCD, although items do not map directly or exclusively onto the four DSM‐5 criteria (Yuan & Dollaghan, 2018). Nevertheless, a single instrument that taps the three domains (language, social pragmatics, autism symptoms) relevant for SPCD diagnosis may be advantageous for clinical evaluation.

### Prevalence

There are currently limited data on SPCD prevalence using DSM‐5 criteria in the general population, nor what functional impacts arise from relatively selective deficits in social‐pragmatic communication (Adams et al., 2015). A South Korean population study of autism prevalence in 7–12 year‐olds reported an SPCD prevalence of 0.5% (Kim et al., 2014). In contrast, in a clinical sample of children referred for autism evaluation, 8% met inclusion criteria for SPCD and the majority of these children had significant behavioural problems (Mandy et al., 2017). However, independent evaluation of structural language skills was not available.

Prevalence has also been estimated in other populations using more broadly defined criteria for social‐pragmatic deficits. Miller et al. (2015) used the Language Use Inventory (LUI; O'Neill, 2007) to compare pragmatic skills in preschoolers with an autistic sibling (*n* = 188) and those without (*n* = 119). Although children who later received an autism diagnosis were excluded, over one‐third of the remaining high‐risk siblings had pragmatic deficits, compared with only 10% in the low‐risk group. Familial risk of autism may therefore increase the risk of SPCD. Furthermore, participants with pragmatic deficits were more likely to have co‐occurring structural language impairment (16% vs. 3% in the group with typical pragmatic development), although the sampling framework may have inflated the association between SPCD and language impairment. Using the original Children's Communication Checklist (CCC; Bishop, 1998), the prevalence of ‘pragmatic language impairment’ in the absence of autism was estimated to be 7.5% of Dutch preschoolers (Ketelaars, Cuperus, van Daal, Jansonius, & Verhoeven, 2009). However, this estimate did not exclude children with structural language impairment, and syntax scores for those with pragmatic language impairment were equivalent to those of children with developmental language disorder. To date, these studies of social‐pragmatic disorder indicate high rates of co‐occurring language and behavioural challenge.

### Functional impairment

Many children with social‐pragmatic deficits have broader social–emotional and behaviour problems (cf. Mandy et al., 2017; Mok et al., 2014) and children identified on the basis of their behavioural problems are frequently found to have both language and social‐pragmatic deficits (Gremillion & Martel, 2014). Others have argued that social‐pragmatic abilities mediate the relationship between structural language and behaviour, especially in areas of socio‐economic disadvantage (Law, Rush, & McBean, 2014). Social‐pragmatic communication skills have also been reported as important school‐readiness skills (Pace, Alper, Burchinal, Golinkoff, & Hirsh‐Pasek, 2019) that account for unique variance in early scholastic success in both reading and maths and development of self‐regulation skills (Ramshook, Welsh, & Bierman, 2020). Not surprisingly, children with SPCD are known to have difficulties with academic skills, particularly reading (Freed, Adams & Lockton, 2015) because of the social cognitive and inferencing skills required to understand text. Thus, research to date indicates that social‐pragmatic deficits are associated with other developmental concerns that increase risk for adverse outcome, underscoring the potential public health costs of SPCD and the need to plan effective health and education services for children with social‐communication deficits.

### Measurement issues

Disentangling pragmatics, structural language and autistic symptomatology have proved challenging. Several studies have sought to evaluate the factor structure of the CCC and CCC‐2 using different statistical techniques (Geurts et al., 2009; Glumbić & Brojčin, 2012; Hawkins, Gathercole, Astle, & Holmes, 2016). A common finding has been the distinction between a structural language factor (speech, syntax and semantics) and a ‘social‐pragmatic’ factor, which combines all nonlanguage subscales (cf. Glumbić & Brojčin, 2012). Geurts et al. (2009) used a combination of Exploratory and Confirmatory Factor Analysis to determine the factor structure of the CCC on an item level. A ‘language form’ factor emerged as separate from pragmatic skills in each analysis; however, CFA indicated poor fit for all models. Hawkins et al. (2016) used exploratory factor analysis on the 10 subscale scores of the CCC‐2 with a clinical sample (*n* = 234) and found two factors, pragmatic and social communication and structural communication, although these were strongly correlated (*r* = .66). None of the previous analyses have revealed a separate factor relating to autism. Instead, Stereotyped Language, Social Relations and Interests subscales are generally subsumed into the social‐pragmatic factor.

Identifying ‘disorder’, be it language or social communication, using cut‐off scores on standardised instruments is equally challenging, with little consensus and variable cut‐offs employed across different studies (Bishop, Snowling, Thompson, & Greenhalgh, 2017). In the CCC‐2, the social‐interaction deviance composite (SIDC) incorporated a difference score to try and identify children with pragmatic deficits relative to structural language skills (Norbury, Nash, Baird, & Bishop, 2004). An alternative is identify children with discrepancies between the two skill profiles, usually operationalised as a 1SD difference between language and pragmatic skills. Cut‐off scores present challenges when measured traits are continuous within the population and the cut points are fairly arbitrary. One goal of the current paper is to determine the functional impact of social‐pragmatic deficits, using both a single cut‐off score and a discrepancy score that identified children with relatively circumscribed social‐pragmatic challenges.

In the current study, we obtained parents and teacher data on a large, geographically defined cohort of children participating in a longitudinal study of language development and disorder (Norbury et al., 2016). Social‐pragmatic skills were assessed when children were aged 5–6 years, at the same time that measures of structural language, cognition and broader social, emotional and behavioural characteristics were obtained. We explicitly test the hypothesis that CCC‐2 subscales load on to three distinct factors representing social‐pragmatic communication, structural language and autistic features. We then identify the percentage of children within this population that present with social‐pragmatic deficits, and the percentage that have these in the absence of language impairment and/or autistic symptoms. We report the functional impacts of social‐pragmatic deficits on social, emotional and behavioural profiles and educational attainment in the first years of primary school. While strict application of DSM‐5 criteria would require exclusion of children with additional language impairments and/or autism, we ask whether the profiles and functional impacts of children with isolated social‐pragmatic deficits differ substantially from those of children with social‐pragmatic deficits that occur in the context of language and social concerns.

## Methods

### Participants

Participants were enrolled in the Surrey Communication and Language in Education Study (SCALES), a prospective, longitudinal study of language developmental and disorder from school entry (Norbury et al., 2016). Initial recruitment targeted all children enrolling in state‐maintained reception classes (263 eligible classrooms, equivalent to US kindergarten) in Surrey, England, during the 2011/12 school year (*n* = 12,398). Teacher report of child communication (Children's Communication Checklist‐Short; CCC‐S, Bishop & Norbury, unpublished), social, emotional and behavioural deficits (Goodman, 1997) and academic progress (Department for Education, 2013) was obtained between May and July 2012 for 7,267 children (6,459 monolinguals) aged 4–5 years (response rate: 61% of all eligible schools and 59% of all eligible children). A stratified random sample of 529 monolingual children was recruited for intensive assessment in Year 1 (age 5–6 years; 83% of invited cohort), with a higher sampling fraction for children with reported low language (40.5% boys, 37.5% girls low language versus 4.3% boys and 4.2% girls typical language).

### Assessment

Teacher ratings of communication in reception year (ages 4–5) were obtained using an online version of the CCC‐S, a 13‐item short form of the Children's Communication Checklist‐2 (Bishop, 2003a). Items are rated on a 4‐point scale and tap children's everyday use of speech, vocabulary, grammar and social discourse; selected items best discriminated clinical cases and controls in a validation study (Norbury et al., 2004), with high degrees of internal consistency (Cronbach's α = .95; Norbury et al., 2016).

Teachers also completed the *Strengths and Difficulties Questionnaire* (SDQ; Goodman, 1997), a well‐validated 25‐item measure of peer relationship problems, hyperactivity/inattention, conduct problems and emotional symptoms, at study intake. Total difficulty scores in the 10th centile (16 or more out of 40) indicated clinically significant social, emotional and behavioural problems. This instrument has demonstrated good psychometric properties (mean Cronbach's α of .73; test–retest estimate of .62, Goodman, 2001), and a specificity of 95% to identify those with psychopathology and sensitivity of 35% to 63% (Goodman, Ford, Simmons, Gatward, & Meltzer, 2003).

Non‐verbal cognitive ability was measured using a composite of block design and matrix reasoning subtests from the *Wechsler Pre‐school and Primary Scales of Intelligence* (WPPSI‐3^rd^ UK edition; Wechsler, 2003).

Language assessment closely followed procedures which have informed DSM‐5 diagnostic criteria for language disorder (Tomblin, Records, Buckwalter, Zhang, & O'Brien, 1997). A total language composite was derived from measures of vocabulary *Receptive and Expressive One‐word Picture Vocabulary Tests* (R/EOWPT; Brownell, 2010), grammar (*Test for Reception of Grammar* (short form); TROG; Bishop (2003b), and *School‐age sentence imitation test‐32 items* SASIT‐32; Marinis, Chiat, Armon‐Lotem, Piper, & Roy, 2011) and narrative *Assessment of Comprehension and Expression: Narrative retelling subtest* (ACE‐Recall; Adams, Cooke, Crutchley, Hesketh, & Reeves, 2001).

Diagnostic information was obtained from teachers, parents and/or the school special educational needs co‐ordinator (SENCO). Access to specialist educational provision was indicated by parent, teacher and/or SENCO report of (i) receipt of a statement of special educational need, the legal document agreeing school placement and additional services required to meet a child's learning needs, (ii) placement in a special education classroom or resource base and (iii) referral to speech‐language therapy services. Expected educational attainment was defined as achieving a ‘good level of development’ at the end of reception year, or achieving all five expected attainments on the scholastic achievement tests (SATS) at the end of Year 2.

The *Children's Communication Checklist‐2* (Bishop, 2003a) was completed by parents and teachers. They rated 70 items by indicating the frequency with which particular communication behaviours occurred on a 4‐point scale (less than once a week, at least once a week, once or twice a day and several times a day). Fifty items were negatively worded (high score implies greater difficulty), and 20 were positively worded, and thus reverse scored. In order to assess the validity of SPCD diagnostic criteria, three new subscales were derived for this measure: a language composite (speech, syntax, semantics and coherence), a social‐pragmatic communication composite (inappropriate initiation, use of context, nonverbal communication) and an autism composite (stereotyped language, interests and social relations). Further description of the CCC‐2 subscale structure and examples is available in Appendix S1.

### Procedure

#### Sampling weights

Sampling weights were constructed as the inverse of the predicted probability of a child being included in the study, so that when weighted, the estimates obtained from the sample are estimates for the whole population. Predicted probabilities of inclusion were estimated via two logistic models; the first logistic model was fitted in the entire population recruited at study intake and included covariates predictive of inclusion due to study design. These were total number of pupils assessed per school and whether the child was identified as ‘high‐risk’ of language disorder (86th centile or above for sex and age group on CCC‐S). The second logistic model was fitted only to children selected for in‐depth assessment and with questionnaire returns. Predictors of participation were tested in a stepwise elimination process and included individual characteristics such as sex, season of birth and IDACI rank score, and school factors such as number of pupils on role, and percentage receiving free school meals (a measure of school‐level deprivation). The final weights were a multiplication of the inverse of the predicted probabilities from the two models.

#### Standardisation of core language measures

Given that many core language tests did not have current or valid UK standardisations, all language and nonverbal composites were standardised using the LMS method (Cole & Green, 1992; Vamvakas, Norbury, Vitoratou, Gooch, & Pickles, 2019). These z‐scores were used to derive five standardised composite scores (vocabulary, grammar, narrative, expressive and receptive language), and the total language composite was formed by averaging the z‐scores of all six direct measures.

#### SCALES language disorder case ascertainment

Children were classified as having either developmental language disorder (DLD), language disorder plus additional diagnosis (LD) or typical language development (TD, see Norbury et al., 2016, for details). DLD was defined as scores of −1.5SD or more on two of the five language composites in the context of no known clinical diagnosis and no intellectual disability. Children with LD met the same language criteria, but had an existing clinical diagnosis (such as autism and Down syndrome) or obtained nonverbal IQ scores of −2SD or more. No formal autism assessment was administered as current diagnostic information from external agencies was obtained from parents and schools, as described above.

### Ethics approval and consent procedures

Consent procedures and study protocol were developed in consultation with Surrey County Council and approved by the Research Ethics Committee at Royal Holloway, University of London, where the study was initiated. Current ethical scrutiny for data management and reporting was obtained from the UCL Research Ethics committee (9322/002). Opt‐out consent was adopted for the first phase as data could be provided anonymously to the research team; 20 families opted‐out. In the second phase, written informed consent for direct assessment was obtained from the parents or legal guardians of all participants and verbal assent was obtained from the children themselves.

#### Parent report sample

**Figure 1:**
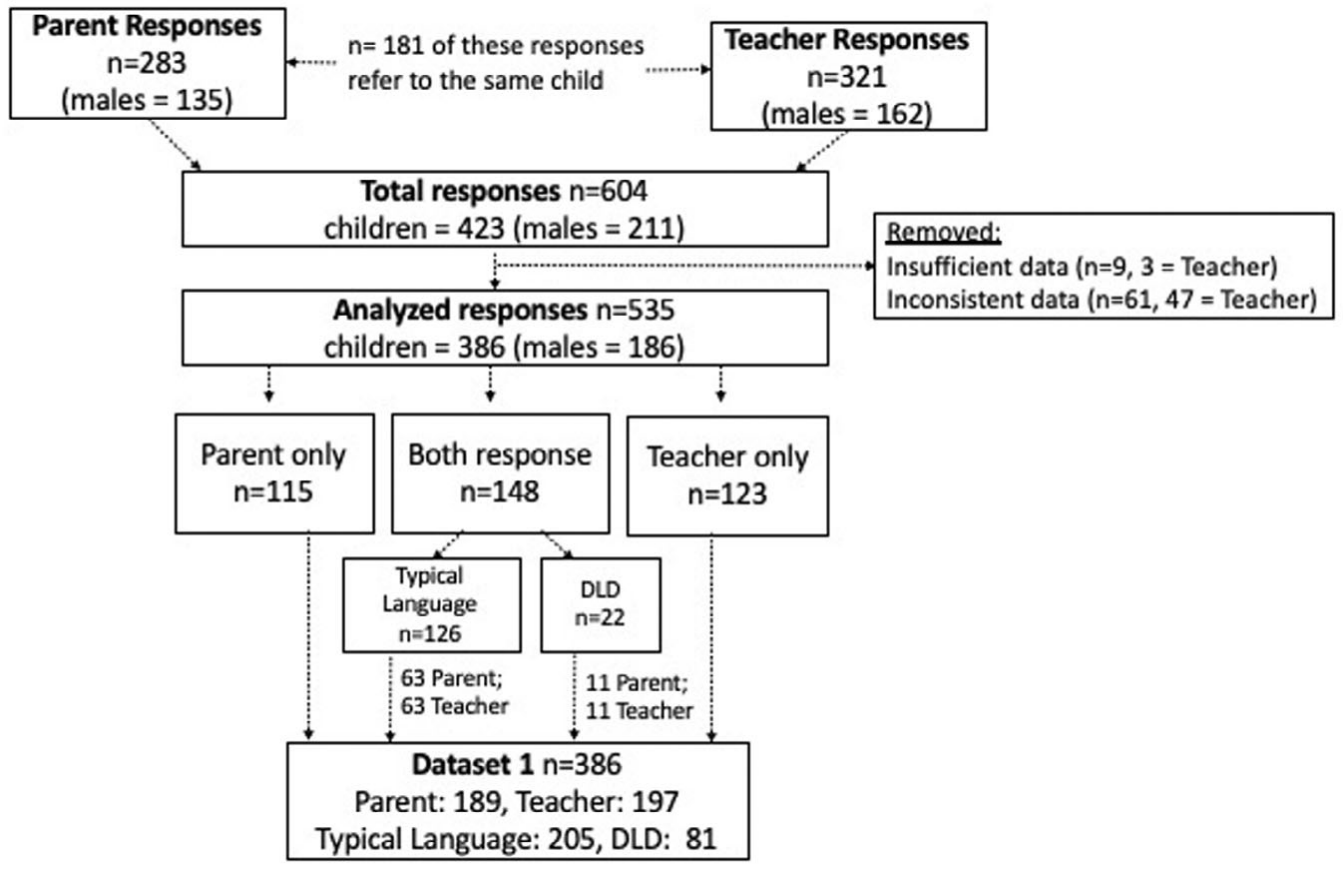
Flowchart representing creation of Dataset 1 (used for CCC‐2 Measurement Model). *Note:* DLD, developmental language disorder

CCC‐2 questionnaires were received for 283 children, as illustrated in Figure 1. Of these, 20 responses were not analysed (six due to insufficiently complete data and 14 due to inconsistencies between negatively and positively worded items). The sample thus comprised 263/529 children (122 males); 143/263 children had been identified as having teacher reported low language at study intake and 52/263 met strict SCALES criteria for language disorder (31 with an existing clinical diagnosis). Responders differed systematically from nonresponders in that they were less likely to have a child that met SCALES criteria for language disorder (χ^2^(*df* = 1) = 8.09, *p* = .004) or have been identified as having teacher reported low language at intake (χ^2^(*df* = 1) = 11.07, *p* < .001). Responders also came from more affluent neighbourhoods (*t*(527) = 4.40, *p* < .001), with children more likely to be female (χ^2^(*df* = 1) = 4.96, *p* = .03).

#### Teacher report sample

Teachers provided CCC‐2 reports for 321 children, as illustrated in Figure 1. Of these, 50 responses were not analysed (three due to insufficiently complete data and 47 due to inconsistencies between negatively and positively worded items). The sample thus comprised 271/529 children (130 males); 143/271 had been identified by their previous teacher as having low language and 51/271 met SCALES criteria for language disorder (24 with an existing clinical diagnosis). There were no significant differences between responders and nonresponders with respect to family socio‐economic status (*t*(527) = 0.25, *p* = .80) or gender (χ^2^(*df* = 1) = 1.47, *p* = .23). As in the parent sample, responders were less likely to describe children meeting DLD criteria (χ^2^ = 6.00, *p* = .01) or those identified at intake as having low language (χ^2^ = 7.72, *p* = .005). Participant characteristics for teacher and parent report samples on demographic and child assessment variables are reported in Table 1; sampling weights that took account of study design were included, so results reflect the population from which this sample was taken.

**Table 1:** Demographic information and performance on key outcome measures reported as mean (SD), [range], % of group in clinical range

	Parent			Teacher		
	Whole sample (*n* = 263)	Typical language (*n* = 211)	Language disorder (*n* = 52)	Whole sample (*n* = 271)	Typical language (*n* = 220)	Language disorder (*n* = 51)
Age (months)	72.3 (5.4) [59–85]	72.3 (5.5) [59–85]	71.9 (4.3) [62–84]	72.8 (4.8) [58–84]	72.8 (4.9) [58–84]	73.2 (3.9) [63–83]
SES (idaci rank)	23,676 (7,529) [3,908–32,459]	24,518 (7,108) [3,908–32,459]	16,683 (7,287) [4,686–31,986]	22,725 (8,014) [4,686–32,471]	23,403 (7,539) [4,686–32,471]	15,342 (9,234) [4,686–31,986]
Ethnicity						
% White	91	92	85	90	91	86
% Mixed/Dual	6	5	8	4	5	4
% Black	<1	0	2	<1	<1	0
% Asian	1	1	2	3	3	2
% Other	2	1	4	3	1	8
Gender						
% Male	46	45	54	48	47	53
Total language (*z*‐score)	0.10 (1.13) [−3.0–2.9]	0.33 (0.94) [−1.4–2.9]	−1.87 (0.48) [−3.0 to −1.2]	0.05 (1.00) [−3.0–2.9]	0.21 (0.87) [−1.5–2.9]	−1.73 (0.51) [−3.0 to −1.0]
NIVQ (*z*‐score)	−0.01 (1.02) [−4.2–2.8]	0.15 (0.93) [−2.2–2.8]	−1.31 (0.73) [−4.2–0.23]	0.06 (0.98) [−2.7–2.8]	0.14 (0.95) [−2.3–2.8]	−0.88 (0.80) [−2.7–1.5]
Total difficulties (SDQ raw score)	5.53 (5.56) [0–27] 14%	5.05 (5.16) [0–24] 9%	9.55 (6.98) [2–27] 35%	5.04 (5.38) [0–32] 13%	4.80 (5.22) [0–26] 10%	7.63 (6.39) [1–32] 25%
Social‐pragmatic CCC‐2 composite	0.32 (0.94) [−2.2–1.9] 11%	0.42 (0.87) [−2.2–1.9] 4%	−0.53 (1.10) [−2.2–1.6] 38%	0.36 (1.06) [−2.7–2.2] 17%	0.47 (0.97) [−2.3–2.2] 10%	−0.86 (1.12) [−2.7–1.8] 47%
Language CCC‐2 composite	0.14 (0.96) [−3.0–1.3] 19%	0.26 (0.87) [−2.5–1.3] 11%	−0.90 (1.06) [−3.0–0.92] 52%	−0.04 (1.12) [−3.3–1.6] 30%	0.11 (0.99) [−2.8–1.6] 20%	−1.60 (1.27) [−3.3–1.3] 73%
Autism CCC‐2 composite	0.23 (0.87) [−2.6–1.6] 8%	0.31 (0.81) [−2.4–1.6] 4%	−0.49 (1.05) [−2.6–1.22] 25%	0.39 (1.01) [−3.0–1.8] 13%	0.48 (0.95) [−2.6–1.8] 9%	−0.56 (1.18) [−3.0–1.6] 31%

### Statistical analysis

All analyses were undertaken in R (R Core Team, 2020) and are weighted unless otherwise specified. CCC‐2 questionnaires were included in analysis if they were at least 90% complete and did not contain gross inconsistencies, defined as a mean raw score difference >1.3 between positively and negatively worded items.

We used confirmatory factor analysis to evaluate the validity of combining subscales to form inclusion (social‐pragmatic communication) and exclusion (language and autism symptoms) latent variables. We preserved the existing subscale structure of the CCC‐2 to facilitate ease of clinical interpretation and application of the instrument. Our dataset for this analysis comprised all children with CCC‐2 data from a single responder (parents: *n* = 115; teachers: *n* = 123). In addition, where both parent and teacher responses were obtained for the same child (*n* = 148), we randomly allocated either the parent or the teacher response to the dataset (intraclass correlation coefficients indicating parent–teacher agreement were moderate: .52 (Autism), .55 (Language) and .62 (Social‐pragmatic), see Appendix S2). These allocations were split evenly across responders and balanced for presence of language disorder (Figure 1).

Model fit was assessed using the Root Mean Square Error Approximation (RMSEA) and Standardized Root Mean Square Residual (SRMR), in both cases values <0.08 indicate good fit; comparative fit index (CFI) and Tucker–Lewis index (TLI), with cut‐offs >0.95 and 0.90, respectively. Identical analyses considered either all teacher responses or all parent responses, with similar results (see Appendix S3). Agreement between parent and teacher reports of symptoms and ‘caseness’ is detailed in Appendix S2, Tables S1 and S2.

Correlations between the language, social‐pragmatic communication and autism composites were weighted. Given the potential for common method bias to inflate correlations between language and social‐pragmatic communication composites derived from the CCC‐2, we also report the correlations between structural language measures based on standardised tests and CCC‐2 subscales and composite scores to evaluate the degree to which structural language and pragmatic skills covary in this population.

The social‐pragmatic composite provided inclusion criteria for SPCD, operationalised in two ways based on previous reports of social‐pragmatic deficit: (a) standardised scores −1SD below the mean, or (b) <10th centile. We acknowledge that cut‐off scores on continuous outcomes are necessarily arbitrary and may yield different patterns of functional impairment. Broader characteristics and educational functional impacts are reported in Table 2. We then estimated how many of these children also met SPCD exclusion criteria. We used a 1SD discrepancy between social‐pragmatic and language composite *z*‐scores to identify individuals with social‐pragmatic deficits relative to language abilities and identified children with an existing clinical diagnosis or scores of −1.5SD or more on the autism composite of the CCC‐2. We also re‐ran these analyses using standardised tests of language (rather than CCC‐2 language composite) as a sensitivity analysis (see Appendix S2, Table S3). Prevalence estimates employed sampling weights to reflect proportions expected in the population sample using (a) teacher, (b) parent, and (c) combined questionnaires. Given the multi‐step branching process described above, estimates are given at each step (see Appendix S2).

**Table 2:** Broader characteristics and educational functional impact of those with score on pragmatic composite below 10th centile reported as mean (SD), [range], % of group in clinical range

	Parent		Teacher	
	Typical pragmatic score (*n* = 234)	Low pragmatic score (*n* = 29)	Typical pragmatic score (*n* = 225)	Low pragmatic score (*n* = 46)
Age (months)	72.3 (5.4) [59–85]	72.5 (4.6) [62–84]	72.7 (4.9) [58–84]	73.6 (3.3) [63–81]
SES (idaci rank)	24,199 (7,184) [3,908–32,459]	15,652 (8,136) [4,686–31,575]	23,135 (7,824) [4,686–32,471]	18,195 (8,663) [6,841–31,986]
% Referred for specialist support	23	90	13	67
Total Language z‐score	0.19 (1.06) [−2.8–2.9]	−1.39 (1.06) [−3.0–0.4]	0.15 (0.96) [−2.8–2.9]	−1.13 (0.65) [−3.0–0.3]
NIVQ z‐score	0.06 (1.00) [−4.2–2.8]	−0.99 (0.73) [−2.7–0.5]	0.11 (0.97) [−2.5–2.8]	−0.55 (0.84) [−2.7–1.1]
Total difficulties (SDQ)	5.11 (5.20) [0–25] 10%	12.00 (6.79) [4–27] 45%	4.44 (4.60) [0–26] 9%	11.66 (8.20) [0–32] 33%
Social‐pragmatic CCC‐2 composite	0.46 (0.80) [−1.2–1.9]	−1.78 (0.34) [−2.2 to −1.3]	0.56 (0.86) [−1.2–2.2]	−1.81 (0.34) [−2.7 to −1.3]
Language CCC‐2 composite	0.24 (0.87) [−2.8–1.3] 13%	−1.39 (1.01) [−3.0–0.8] 69%	0.15 (0.94) [−2.8–1.6] 19%	−2.16 (0.63) [−3.3 to −0.3] 87%
Autism CCC‐2 composite	0.35 (0.74) [−1.7–1.6] 2%	−1.70 (0.57) [−2.6 to −0.1] 59%	0.59 (0.79) [−1.6–1.8] 2%	−1.78 (0.60) [−3.0 to −0.2] 67%

## Results

### Measurement model

**Figure 2:**
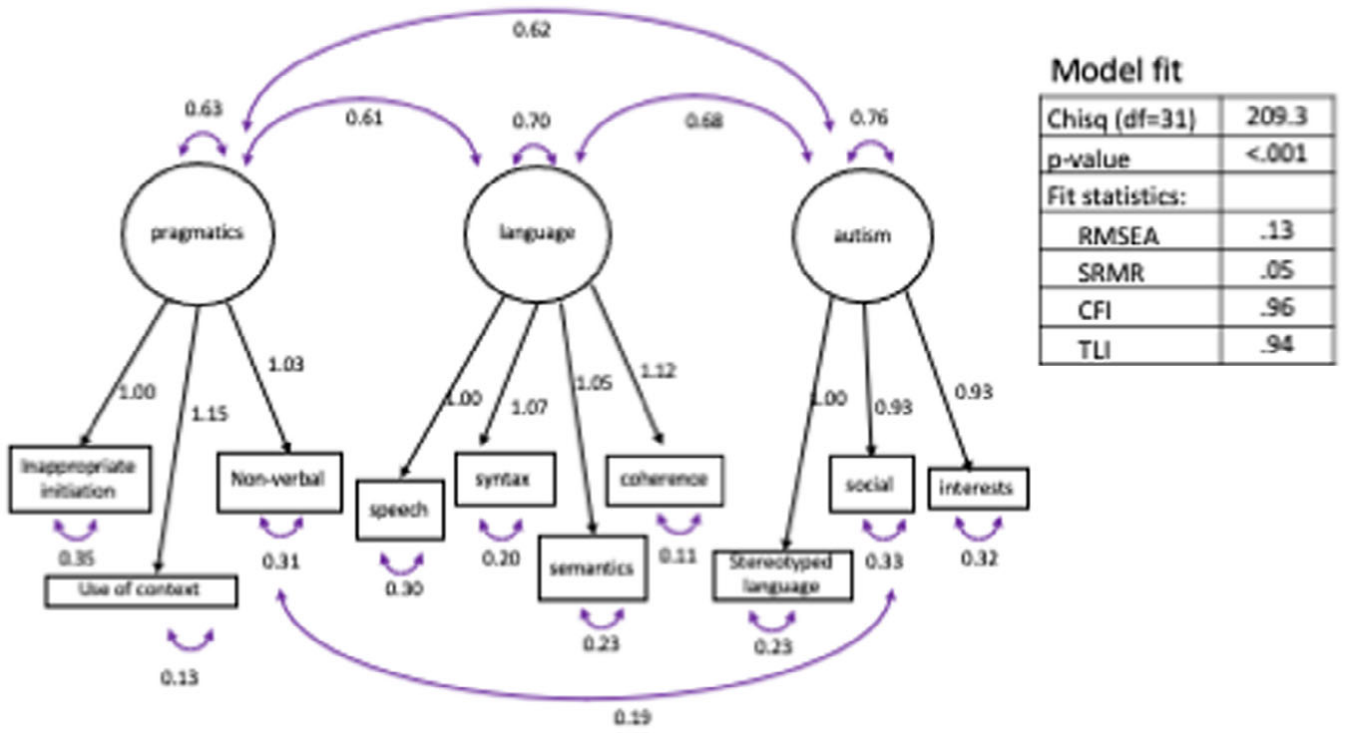
Three factor measurement model and fit statistics for CCC‐2 subscale data using Dataset 1. *Note:* CFI, comparative fit index; RMSEA, root mean square error approximation; SRMR, standardized root mean square residual; TLI, tucker lewis index [Color figure can be viewed at wileyonlinelibrary.com]

The hypothesised 3‐factor solution (language, social‐pragmatic, autism, Figure 2) was an adequate fit to the data and was a significantly better fit than a two‐factor (Figure S1; χ^2^(*df* = 2) = 29.7, *p* < .001) or one‐factor model (Figure S2; χ^2^(*df* = 3) = 229.7, *p* < .001). The same analysis using teacher and parent samples separately yielded similar results (see Appendix S3, Tables S4–S6 for fit statistics for each model).

**Figure 3:**
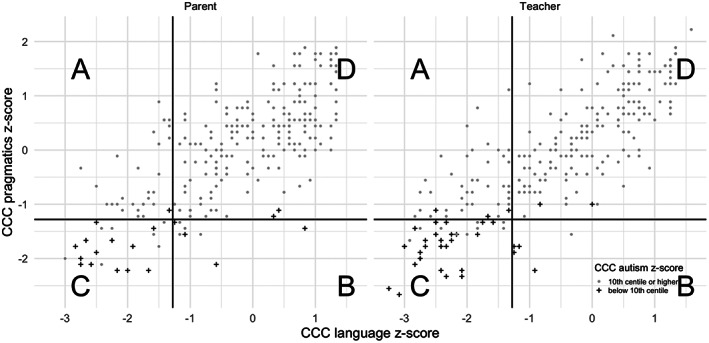
CCC‐2 language, pragmatics and autism composite z‐scores. *Note:* Participants are allocated to quadrants A‐D according to their CCC‐2 language and social‐pragmatic communication composites; Quadrant A indicates a primarily structural language deficit (DLD), Quadrant B indicates a primarily social communication deficit (SPCD), Quadrant C indicates deficits in both domains and Quadrant D indicates no deficit in either domain. Line = 10th centile cut‐off

Each factor composite score had excellent internal consistency, with Cronbach's α ranging from .93 to .97 (teacher sample) and .92 to .97 (parent sample). The three composites were highly correlated with one another (*r*s = .75–.88 teachers; .68–.86 parents) and moderately correlated with total language composite based on standard assessments (*r*s = .28–.54 teachers; .33–.53 parents, see Table S7). Figure 3 demonstrates the relationship between the three CCC‐2 composites for both parent (Panel 1) and teacher (Panel 2) data.

### Identifying cases of SPCD

Cut‐off scores of 10th centile below the mean were used to identify children with low language but not low social‐pragmatic communication scores (low language‐only, quadrant A of Figure 3), low social‐pragmatic communication but not language scores (low pragmatics‐only, quadrant B), below cut‐off on both (low language and pragmatics, quadrant C) or neither (typical language and pragmatics, quadrant D). Additionally, children scoring ‐10th centile below the mean on the autism composite are indicated by plus markers.

### Prevalence of SPCD

We estimated the population prevalence of SPCD using sampling weights. In total, teachers reported that 8% of the mainstream school population at Year 1 scored below the 10th percentile on the social‐pragmatic composite, similar to parent report of 6%.

However, the majority of cases identified using these cut‐off scores had concomitant deficits in structural language. We therefore used discrepancy scores to identify children with disproportionate social‐pragmatic impairments relative to structural language skills. Only 0.7% (teacher data) and 1.4% (parent data) had social‐communication deficits disproportionate (greater than 1SD difference) to structural language abilities. Furthermore, children with social‐communication deficits had more autistic symptoms than peers (parent sample: *t*(259) = 10.6, *p* < .001; teacher sample: *t*(266) = 13.8, *p* < .001). In the teacher sample, 30 of the 46 children were male, 31 had an existing clinical diagnosis and 24 met SCALES criteria for language disorder (−1.5SD on 2/5 language composite scores). Only one child met all exclusion criteria for SPCD. The parent sample yielded similar results: 19 of 29 children were male, 26 had a clinical diagnosis and 20 met SCALES criteria for language disorder, and only two met all inclusion/exclusion criteria for SPCD. Thus, if exclusionary criteria are applied, the population prevalence is estimated to be <1%.

### Functional impact

Children with social‐pragmatic deficits (<10th centile) were compared with peers on a range of cognitive, academic and behavioural measures (Table 2). These children were more likely to have poorer academic attainment (Early Years Foundation Stage Profile: χ^2^(*df* = 1) = 24.2 (teacher), 20.8 (parent); SATS: χ^2^(*df* = 1) = 26.2 (teacher), 30.2 (parent), all *p*s < .001), and have total difficulties scores on the SDQ in the clinical range (parent: χ^2^(*df* = 1) = 23.9, *p* < .001; teacher: χ^2^(*df* = 1) = 16.0, *p* < .001). We also compared children in each quadrant of Figure 3 to determine whether children with combined language and pragmatic deficits had more severe behaviour and academic outcomes relative to peers with relatively circumscribed deficits in either domain (with the caveat that numbers in those cells are small). Here, we find no significant differences in academic outcomes for members of quadrants A, B and C, however quadrant A (Language‐only deficit) children had significantly lower behavioural difficulties symptom scores than those with difficulties in both domains (quadrant C), whereas those in quadrant B (SPCD profile) either did not differ (parents) or actually scored higher (teacher sample, see Appendix S4). Finally, given that our analyses suggest that social‐pragmatic deficits may be best conceptualised as a continuum of skill rather than a discrete diagnostic group, we conducted regression analyses to estimate the influence of social‐pragmatic skill on behaviour and academic outcomes, after accounting for language, socio‐economic status and nonverbal cognitive ability (note that we could not include autism symptoms as a predictor variable due to multicollinearity with the social‐pragmatic score). Social‐pragmatic score on the CCC‐2 was a unique predictor of total behaviour scores on the SDQ and contributed unique variance to likelihood of good level of development in EYFS assessment in addition to grammar. It did not, however, predict unique variance in Year 2 SATS achievement after accounting for structural language (see Appendix S5, Table S8).

## Discussion

This study aimed to estimate the prevalence and functional impact of social‐pragmatic deficits in a population‐derived sample. Our major finding is that children presenting with ‘pure’ SPCD were extremely rare (<1% estimated population prevalence). Instead, social‐pragmatic deficits were commonly associated with structural language and behavioural deficits and subthreshold autism symptoms. Together, this profile yielded significant, negative impacts on academic attainment in the early school years. Regression analyses further indicated that pragmatics and language make independent contributions to general behaviour and academic outcomes.

Our prevalence estimates align with a population study of South Korean 7–12 year‐olds which reported SPCD prevalence of 0.5% (Kim et al., 2014). Those identified as having social‐pragmatic deficits were a heterogenous group, and 29% would have met previous DSM‐4 diagnostic criteria for pervasive developmental disorder‐not otherwise specified (PDD‐NOS), a diagnosis typically given when an individual experiences some features of autism but does not meet the threshold for an autism diagnosis. Similarly, Mandy et al. (2017) characterised many of their SPCD sample as meeting autism social‐communication criteria but not RRBI. None of these studies has reported the temporal stability of social‐pragmatic skills or the stability of SPCD diagnosis, so a follow‐up of this cohort would be highly informative.

Although very few children in this sample would meet all DSM‐5 criteria for a SPCD diagnosis, a significant number of children (6–8% at 10th centile) presented with poor social‐pragmatic skills. This echoes previous findings: Ketelaars et al. (2009) identified 7–8% of 4‐year‐olds in a community sample with pragmatic difficulties and Miller et al. (2015) found pragmatic difficulties in 10% of 3‐year‐olds. Children with social‐pragmatic deficits are, however, also likely to have challenges across a broad range of developmental domains. In our sample, 69–87% had structural language difficulties and 33–45% met clinical thresholds for behavioural difficulties. Others have documented the high comorbidity between social‐pragmatic deficits and behavioural difficulties and their likely bidirectional relationship. For example, inattention and hyperactivity may prevent a child from using social‐pragmatic skills effectively (e.g. waiting turn in conversation and noticing nonverbal communicative cues). Equally, poor social pragmatics can interfere with peer relationships and behavioural regulation (Hawkins et al., 2016). Social‐pragmatic deficits precede and predict behaviour problems (Snowling, Bishop, Stothard, Chipchase, & Kaplan, 2006; St Clair, Pickles, Durkin, & Conti‐Ramsden, 2011) and may mediate the relationship between structural language and behaviour (Helland, Lundervold, Heimann, & Posserud, 2014; Law et al., 2014). Future research could employ intervention designs to elucidate the causal relationships between language, social‐pragmatic skill and behaviour, with implications for shaping developmental trajectories.

We hypothesised that the CCC‐2 would be an appropriate tool to measure the inclusion and exclusion criteria for SPCD. Our measurement model broadly supported the proposed three‐factor model of the CCC‐2 (language, social pragmatics and autism), indicating that while strongly correlated, language, social‐pragmatic skills and autistic features can be delineated to some extent. Gibson, Adams, Lockton, and Green (2013) found that children with pragmatic language impairment (who may meet DSM‐5 SPCD criteria) and those with autism were distinguishable from one another on measures of restricted and repetitive behaviours and interests, peer relations and expressive language. Other investigations have highlighted that some children meet DSM‐5 autism criteria for social communication but do not have RRBIs and thus qualify for a SPCD diagnosis (Mandy et al., 2017; Mandy, Charman, Gilmour, & Skuse, 2011). However, it is clear that these skills overlap and are better conceptualised as a continuum rather than distinct domains. Future consideration of how subthreshold symptoms in multiple domains increase risk, or whether increased skill in one‐factor domain increases resilience in the face of social‐pragmatic deficit will be necessary to optimise intervention approaches.

This study demonstrated how the CCC‐2 could be an accessible tool to examine inclusion and exclusion criteria for SPCD in children. In the current study, the structural language composite demonstrated high correlation with directly measured language skills, confirming that the CCC‐2 is a valid observer report measure of structural language. There is currently no agreed gold‐standard instrument with which to directly measure social‐pragmatic skills (Timler & Covey, 2021; Visser & Tops, 2017), making it challenging to assess the validity of the CCC‐2 as a measure of pragmatic language. The lack of validated measure of pragmatics is one of the many challenges of the new SPCD diagnosis. New experimental measures that require pragmatic responses in real‐time show promise, but their diagnostic accuracy requires further investigation (Wilson & Bishop, 2022). Since developing, evaluating and norming new instruments is expensive and time consuming, the CCC‐2 is a good starting point for future studies. Further specificity could be obtained by either combining items from the CCC‐2 with items from other scales (Mandy et al., 2017) or employing item response theory (Topal, Samurcu, Taskiran, Tufan, & Semerci, 2018; Yuan & Dollaghan, 2018, 2020); however, attempts to do so have reported poor agreement between professionals about how questionnaire items map onto DSM‐5 criteria (Yuan & Dollaghan, 2018).

### Strengths and limitations

A strength of this study is that for a subset of the children sampled, teacher and parent‐reported pragmatic skills could be directly compared and inter‐rater reliability was found to be moderate**.** Inter‐rater differences are thought to arise not only because teachers and parents observe an individual's behaviour in different settings but also due to differing expectations, reference points and motivations (Richters, 1992; Sims & Lonigan, 2012; Tripp, Schaughency, & Clarke, 2006). Geurts et al. (2004) found that using both responses on the CCC led to a slight improvement in child classification. This suggests that for investigations of prevalence and correlation, either respondent would suffice but on an individual level when seeking accurate diagnosis, a holistic approach, taking into account perspectives of both parents and teachers is preferable.

Some limitations of this study should be acknowledged. First, the pattern of questionnaire return, particularly in the parent sample, was biased towards higher SES and lower rate of identified language difficulties. We addressed this issue through the use of sampling weights, but it remains possible that these underestimate true population prevalence. Second, it was not possible to independently assess autism symptoms, or directly measure restricted and repetitive behaviours; it is therefore possible that our sample includes undiagnosed autistic children that have been misclassified. If this was the case, the SPCD prevalence estimate would be even lower. Third, while promising, the CCC‐2 is not a diagnostic instrument and diagnosis on the basis of one assessment is not recommended. Triangulation with interview, observation and direct assessment of a range of social‐pragmatic skills is advocated. Finally, like previous analyses, we found that the proposed factor structure did not fit the data terribly well, due largely to the high correlations between latent variables and significant cross‐loadings amongst subscales (Ash, Redmond, Timler, & Kean, 2017; Geurts et al., 2009). Rather than being an artefactual feature of the measurement tool, this may reflect the true picture of intertwined competencies which make up our communicative ability.

## Conclusions

The CCC‐2 is a reasonable tool for identifying children who may require additional support with one or more aspects of communication. A considerable proportion of children in the early years of primary school have social‐pragmatic deficits that interfere with social and scholastic activity. However, it is rare that children experience these deficits in isolation. The exclusionary criteria of DSM‐5 therefore represent a false dichotomy that may lead to underidentification of individuals with striking social‐pragmatic deficits that could benefit from tailored support and intervention. A benefit of the new diagnosis could be to focus therapeutic efforts on social‐pragmatic skills to determine which are malleable and result in improved functional outcomes. Our findings suggest that such interventions should avoid narrow focus and incorporate elements of structural language, and should test cascading impacts on other aspects of development, such as behaviour and emotion regulation.

## Supporting information


**Figure S1.** Two‐factor measurement model and fit statistics for CCC‐2 subscale data using Dataset 1.

**Figure S2.** One‐factor measurement model and fit statistics for CCC‐2 subscale data using Dataset 1.

**Appendix S1.** CCC‐2 Subscales.
**Appendix S2.** Parent‐Teacher agreement.
**Appendix S3.** Measurement model.
**Appendix S4.** Functional impact additional group comparisons.
**Appendix S5.** Functional impact additional regression analyses.
**Table S1.** Inter‐rater reliability and prevalence for different operationalisations of SPCD.
**Table S2.** Inter‐rater reliability and prevalence for different operationalisations of SPCD using SIDC.
**Table S3.** Inter‐rater reliability and prevalence for different operationalisations of SPCD using direct language measures.
**Table S4.** Model fit characteristics for CFA (dataset 1).
**Table S5.** Model fit characteristics for CFA using Teacher data.
**Table S6.** Model fit characteristics for CFA using Parent data.
**Table S7.** Correlation between CCC‐2 Subscales, Composites and Language Scores.
**Table S8.** Linear regression models predicting SDQ total difficulties scores, EYFS screening outcomes and achieving all five SATS attainments.
