# Supplementary figures and images for: Prevalence and functional impact of social (pragmatic) communication disorders
Source: J Child Psychol Psychiatry. 2022 Sep 16;64(3):376–87. doi: 10.1111/jcpp.13705 (PMC10087005; doi:10.1111/jcpp.13705)

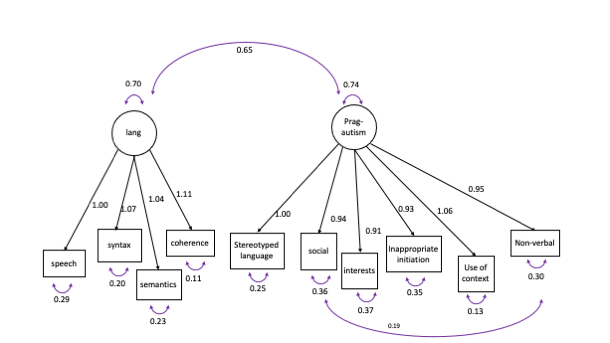

Supplement: Supplementary file 1 — Figure S1. Two‐factor measurement model and fit statistics for CCC‐2 subscale data using Dataset 1. [file JCPP-64-376-s001.png]

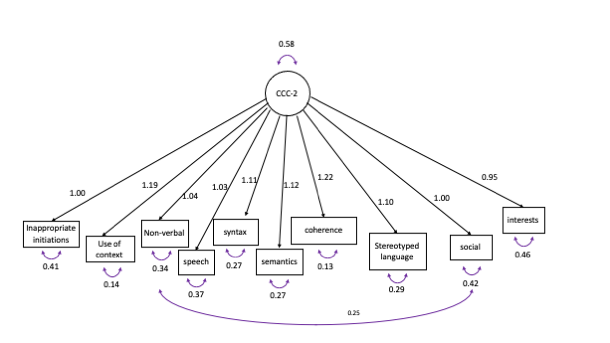

Supplement: Supplementary file 2 — Figure S2. One‐factor measurement model and fit statistics for CCC‐2 subscale data using Dataset 1. [file JCPP-64-376-s002.png]
