# Supplementary material for: Prevalence and functional impact of social (pragmatic) communication disorders
Source: J Child Psychol Psychiatry. 2022 Sep 16;64(3):376–87. doi: 10.1111/jcpp.13705 (PMC10087005; doi:10.1111/jcpp.13705)
Supplement: Supplementary file 3 — Appendix S1. CCC‐2 Subscales. Appendix S2. Parent‐Teacher agreement. Appendix S3. Measurement model. Appendix S4. Functional impact additional group comparisons. Appendix S5. Functional impact additional regression analyses. Table S1. Inter‐rater reliability and prevalence for different operationalisations of SPCD. Table S2. Inter‐rater reliability and prevalence for different operationalisations of SPCD using SIDC. Table S3. Inter‐rater reliability and prevalence for different operationalisations of SPCD using direct language measures. Table S4. Model fit characteristics for CFA (dataset 1). Table S5. Model fit characteristics for CFA using Teacher data. Table S6. Model fit characteristics for CFA using Parent data. Table S7. Correlation between CCC‐2 Subscales, Composites and Language Scores. Table S8. Linear regression models predicting SDQ total difficulties scores, EYFS screening outcomes and achieving all five SATS attainments. [file JCPP-64-376-s003.docx]

**Supporting Information**

**Appendix S1.** CCC-2 Subscales

The original CCC comprised 9 subscales, which under the CCC-2 were amended to the following 10 subscales, each with seven items (example of each provided in italics):

1. Speech (*pronounces words in a babyish way, such as ‘chimbley’ for ‘chimney’ or ‘bokkle’ for ‘bottle’*);
2. Syntax (*gets mixed up between he/him or she/her, so might say ‘him is working’ rather than ‘he is working’ or ‘her have a cake’ rather than ‘she has a cake’*);
3. Semantics (*mixes up words of similar meaning, e.g. might say ‘dog’ for ‘fox’ or ‘screwdriver’ for ‘hammer’*);
4. Coherence (*can be hard to tell if s/he is talking about something real or make-believe*);
5. Inappropriate initiation (*talks repeatedly about things that no-one is interested in*);
6. Stereotyped language (*repeats back what others have just said. For instance, if you ask ‘what did you eat?’ might say, ‘what did I eat?’*);
7. Use of context (*misses the point of jokes and pubs (though may be amused by nonverbal humour such as slapstick*);
8. Non-verbal communication (*does not look at the person s/he is talking to*);
9. Social relations (*appears anxious in the company of other children*);
10. Interests (*talks about lists of things s/he has memorised e.g. the names of the capitals of the world, or the names of varieties of dinosaurs*).

Sum scores for each subscale were transformed into age-adjusted scaled scores with a mean of 10 (SD = 3), based on the published norms.^13^ The CCC-2 contains two summary scores: the General Communication Composite (GCC) and the Social Interaction Deviance Composite (SIDC). The GCC sums the first eight sub-scales (excluding Interests and Social Relationships, which were designed to tap symptoms of autism spectrum disorder); a GCC score of 55 or below (10th centile) indicates poor communication and has been sensitive to the communication challenges of autistic individuals who score within the normal range on standardised tests of language structure (Norbury et al. 2004). The SIDC is calculated by subtracting standardized scores on Scales A, B, C and D from those on Scales E, H, I and J. It seeks to identify children with disproportionate social-pragmatic deficits relative to overall structural language ability. SIDC scores had acceptable levels of parent-teacher agreement in the validation sample.^26^

**Appendix S2.** Parent-Teacher agreement

Intra-Class Correlation (ICC)

Parent-teacher agreement for each composite is reported as the intra-class correlation co-efficient for 148 children (66 males) for whom both questionnaires were returned. The corresponding coefficients can be interpreted as follows: <.40 are poor, .40-.59 fair, .60-.74 good and .75-1.00 excellent (Cicchetti, 1994). Intra-class correlation co-efficients were .55, .62 and .52 for Language, Pragmatic and Autism composites respectively. Inter-rater agreement was lower for children identified as meeting Developmental Language Disorder criteria (.25, .34 and .22 respectively for Language, Social-pragmatic and Autism sub-scales, compared with .47, .61 and .54 in the Typically Developing group). ICC for the General Communication Composite (GCC) and Social Interaction Deviance Composite (SIDC) were .56 and .42.

Caseness

For those children with both teacher and parent questionnaires, Kappa statistics report the extent to which responders agree on ‘caseness’ using a given set of criteria. Cohens Kappa can be interpreted as follows: < 0 no agreement, .0–.20 poor, 0.21–0.40 fair 0.41–0.60 moderate, 0.61–0.80 substantial and 0.81–1 almost perfect agreement (Landis & Koch, 1977). Agreement on ‘caseness’ for children with low social-pragmatic skills (either using the 1SD or 10^th^ centile cut-off) is shown in Tables S1 and S2, as well as for the various operationalisations of SPCD. Using SIDC and GCC to identify those with disproportionate pragmatic difficulties to their language skills yielded low agreement (.42 at 1SD and .23 at 10^th^ centile).

Agreement on presenting difficulty

We used the approach outlined in Figure 3 to allocate children to Quadrant A, B, C or D depending on their profile of CCC-2 language and social pragmatic scores, for the 146 children with both complete parent and teacher reports. We found that respondents agreed on 112 classifications. Almost all of these were agreements of typical development (100/112), with minimal agreement on the nature of the presenting difficulty (12/46). The resulting Kappa statistic suggested minimal agreement on ‘caseness’ (κ = .42).

**Table S1.** Inter-rater reliability and prevalence for different operationalisations of SPCD

| **Criterion** | **inter-rater reliability**  **(n=148)** | using teacher sample (n=271) | | using parent sample (n=263) | | Using dataset1 (n=386) | |
| --- | --- | --- | --- | --- | --- | --- | --- |
|  |  | **cases** | **weighted prev** | **cases** | **weighted prev** | **cases** | **weighted prev** |
| **low social-pragmatic skills (-1SD)** | .55 | 57 | 10.4% | 45 | 9.7% | 77 | 10.5% |
| remove those with > 1SD diff between language and social pragmatic z-score | .66 | 2 | 0.7% | 4 | 2.0% | 5 | 1.6% |
| Remove those with Intellectual Disability, autism or other diagnoses | NA | 1 | 0.6% | 2 | 1.3% | 2 | 0.9% |
| Remove those with < -1.5SD on autism CCC-2 composite | NA | 0 | 0% | 0 | 0% | 0 | 0% |
|  | | | | | | | |
| **low social-pragmatic skills (10th centile)** | .54 | 46 | 8.3% | 29 | 6.1% | 57 | 7.3% |
| remove those with > 1SD diff between language and social pragmatic z-score | .01 | 2 | 0.7% | 2 | 1.4% | 4 | 1.5% |
| Remove those with Intellectual Disability, autism or other diagnoses | NA | 1 | 0.6% | 1 | 0.7% | 2 | 0.9% |
| Remove those with < -1.5SD on autism CCC-2 composite | NA | 0 | 0% | 0 | 0% | 0 | 0% |

**Table S2.** Inter-rater reliability and prevalence for different operationalisations of SPCD using SIDC

| **Criterion** | **inter-rater reliability**  **(n=148)** | using teacher sample (n=271) | | using parent sample (n=263) | | Using dataset1 (n=386) | |
| --- | --- | --- | --- | --- | --- | --- | --- |
|  |  | **cases** | **weighted prev** | **cases** | **weighted prev** | **cases** | **weighted prev** |
| **low social-pragmatic skills (-1SD)** | .55 | 57 | 10.4% | 45 | 9.7% | 77 | 10.5% |
| remove those with SIDC < -15 or negative SIDC AND GCC < = 55 | .42 | 6 | 2.8% | 11 | 4.2% | 11 | 3.9% |
| Remove those with Intellectual Disability, autism or other diagnoses | NA | 1 | 0.6% | 3 | 1.4% | 3 | 1.0% |
| Remove those with < -1.5SD on autism CCC-2 composite | NA | 0 | 0% | 1 | 0.1% | 1 | 0.1% |
|  | | | | | | | |
| **low prag (10th centile)** | .54 | 46 | 8.3% | 29 | 6.1% | 57 | 7.3% |
| remove those with SIDC < -15 or negative SIDC AND GCC < = 55 | .23 | 6 | 2.8% | 8 | 2.9% | 9 | 3.5% |
| Remove those with Intellectual Disability, autism or other diagnoses | NA | 1 | 0.6% | 2 | 0.8% | 2 | 0.7% |
| Remove those with < -1.5SD on autism CCC-2 composite | NA | 0 | 0% | 1 | 0.1% | 1 | 0.1% |

In order to sensitivity check our finding that prevalence of isolated social communication difficulties was very low, we re-ran the analysis presented under Prevalence of SPCD using direct measures of grammar and vocabulary only. Thus, rather than using a structural language measure derived from the CCC-2 composite (either SIDC or 1SD differential between structural and pragmatic composite) to identify children with concomitant language disorder (thus excluded from SPCD diagnosis), we used directly measured grammar and vocabulary scores. This resulted in similarly low prevalence levels.

As before, we included all children scoring 1SD below the mean on the CCC-2 pragmatic composite. Next, we excluded all those who scored an average of 1SD below the mean on direct measures of expressive and receptive grammar and vocabulary (combined).

We then excluded those with an additional clinical diagnosis, and further excluded those with autism symptoms (CCC-2 autism composite greater than 1.5 SD below the mean). We carried out the same procedure including only those scoring below the 10^th^ centile on the CCC-2 pragmatics composite.

**Table S3.** Inter-rater reliability and prevalence for different operationalisations of SPCD using direct language measures

| **Criterion** | **inter-rater reliability**  **(n=148)** | using teacher sample (n=271) | | using parent sample (n=263) | | Using dataset1 (n=386) | |
| --- | --- | --- | --- | --- | --- | --- | --- |
|  |  | **cases** | **weighted prev** | **cases** | **weighted prev** | **cases** | **weighted prev** |
| **low social-pragmatic skills (-1SD)** | .55 | 57 | 10.4% | 45 | 9.7% | 77 | 10.5% |
| remove those with > 1SD below mean on combined vocabulary and grammar z score | NA | 22 | 5.1% | 19 | 5.4% | 29 | 5.2% |
| Remove those with Intellectual Disability, autism or other diagnoses | NA | 10 | 1.9% | 8 | 2.8% | 13 | 2.4% |
| Remove those with < -1.5SD on autism CCC-2 composite | NA | 5 | 1.0% | 6 | 1.5% | 7 | 1.2% |
|  | | | | | | | |
| **low social-pragmatic skills (10th centile)** | .54 | 46 | 8.3% | 29 | 6.1% | 57 | 7.3% |
| remove those with > 1SD below mean on combined vocabulary and grammar z score | NA | 18 | 4.2% | 8 | 2.4% | 20 | 3.4% |
| Remove those with Intellectual Disability, autism or other diagnoses | NA | 7 | 1.0% | 3 | 0.9% | 9 | 1.3% |
| Remove those with < -1.5SD on autism CCC-2 composite | NA | 2 | 0.2% | 2 | 0.2% | 3 | 0.2% |

References

﻿Cicchetti, D.V. (1994). Guidelines, criteria, and rules of thumb for evaluating normed and standardized assessment instruments in psychology. *Psychological Assessment, 6,* 284–290.

Landis, J. R. & Koch, G. G. (1977). ﻿The measurement of observer agreement for categorical data. *Biometrics, 33*, 159–174.

**Appendix S3.** Measurement model

We used ‘Dataset 1’ (illustrated in Figure 1) to compare and evaluate three CFA models:

- Our hypothesised Three factor model with latent variables representing a Language composite (Speech, Syntax, Semantics and Coherence sub-scales), a Social-Pragmatic Communication composite (Inappropriate initiation, Use of Context, Non-verbal Communication sub-scales) and an Autism symptom composite (Stereotyped language, Interests, and Social Relations sub-scales, see Figure 2).
- A simpler Two factor model where all four language subscales (Speech, Syntax, Semantics and Coherence) load onto a structural language latent variable, and the remaining six subscales load on to an ‘social pragmatic-autism’ latent variable (Figure S1)
- An even simpler One factor model where all 10 subscale indicators load onto one latent variable (Figure S2)

Model comparison used Chi-square analysis and we considered the following fit statistics when evaluating each model: Root Mean Square Error Approximation (RMSEA) and Standardized Root Mean Square Residual (SRMR), in both cases where a value closer to zero indicates a good fit, with a cut-off of 0.08; Comparative Fit Index (CFI) and Tucker Lewis Index (TLI), which both compare the target model to a null model and improvement is indicated by a value close to 1, with cut-offs of 0.95 and 0.90 respectively.

We encountered model convergence issues and consulted modification indices to resolve them, resulting in an additional covariance parameter between nonverbal and social subscales being added to every model.

The three factor model is statistically preferable to the 2 and 1 factor solutions, but the fit indices suggest adequate-poor model fit (see Table S4).

Identical analyses were also run using either all teacher responses or all parent responses.

Teacher Responses

The hypothesised 3 factor solution was evaluated using Confirmatory Factor Analysis for the teacher sample only. It was a significantly better fit to the data than the two-factor model (χ^2^(df=2)=52.6, p<.001). The two- factor model was itself a significantly better fit than the one-factor model (χ^2^ (df=1)=230.8, p<.001). Fit statistics for each model are presented in Table S5.

Parent Responses

Similarly, the three-factor solution was evaluated using Confirmatory Factor Analysis for the parent sample only. It was not a significantly better fit to the data than the two-factor model (χ^2^ (df=2)=5.1, p=.078). Both the three- and two-factor models were a significantly better fit than the one-factor model (χ^2^ (df=1)=130.5, p<.001 and χ^2^ (df=1)=125.4, p<.001). Fit statistics for each model are presented in Table S6.

**Table S4.** Model fit characteristics for CFA (dataset 1)

| Model | 1 | 2 | 3 |
| --- | --- | --- | --- |
| Latent variables | 3  (three-factor) | 2  (two-factor) | 1  (one-factor) |
| Df | 31 | 33 | 34 |
| Chisq | 209.3 | 238.9 | 439.0 |
| p-value | *** | *** | *** |
| Fit statistics: |  |  |  |
| RMSEA | 0.12 | 0.13 | 0.18 |
| SRMR | 0.05 | 0.05 | 0.07 |
| CFI | 0.96 | 0.95 | 0.90 |
| TLI | 0.94 | 0.93 | 0.87 |

**Table S5.** Model fit characteristics for CFA using Teacher data^[[1]](#footnote-1)^

| Model | 1 | 2 | 3 |
| --- | --- | --- | --- |
| Latent variables | 3  (three-factor) | 2  (two-factor) | 1  (one-factor) |
| Df | 30 | 32 | 33 |
| Chisq | 170.7 | 219.5 | 352.7 |
| p-value | *** | *** | *** |
| Fit statistics: |  |  |  |
| RMSEA | 0.13 | 0.15 | 0.19 |
| SRMR | 0.04 | 0.05 | 0.07 |
| CFI | 0.96 | 0.94 | 0.90 |
| TLI | 0.93 | 0.92 | 0.86 |

**Table S6.** Model fit characteristics for CFA using Parent data

| Model | 1 | 2 | 3 |
| --- | --- | --- | --- |
| Latent variables | 3  (three-factor) | 2  (two-factor) | 1  (one-factor) |
| Df | 31 | 33 | 34 |
| Chisq | 158.6 | 166.2 | 270.9 |
| p-value | *** | *** | *** |
| Fit statistics: |  |  |  |
| RMSEA | 0.13 | 0.12 | 0.16 |
| SRMR | 0.05 | 0.05 | 0.07 |
| CFI | 0.95 | 0.94 | 0.90 |
| TLI | 0.92 | 0.92 | 0.87 |

**Figure S1**

Two factor measurement model and fit statistics for CCC-2 subscale data using Dataset 1.

**Figure S2**

One factor measurement model and fit statistics for CCC-2 subscale data using Dataset 1

**Table S7:** Correlation between CCC-2 Subscales, Composites and Language Scores

|  | **CCC-2 Subscales** | | | | | | | | | |  | **CCC-2 Subscales** | | |  | **Language Z scores** | | |
| --- | --- | --- | --- | --- | --- | --- | --- | --- | --- | --- | --- | --- | --- | --- | --- | --- | --- | --- |
|  | 1 | 2 | 3 | 4 | 5 | 6 | 7 | 8 | 9 | 10 |  | 11 | 12 | 13 |  | 14 | 15 | 16 |
| **CCC-2 Subscales** | | | | | | | | | | | | | | | | | | |
| 1. Speech |  | .65 | .57 | .55 | .32 | .49 | .41 | .36 | .31 | .33 |  | .42 | .83 | .45 |  | .43 | .42 | .42 |
| 2. Syntax | .72 |  | .58 | .59 | .46 | .54 | .50 | .48 | .39 | .38 |  | .55 | .85 | .52 |  | .47 | .51 | .50 |
| 3. Semantics | .60 | .68 |  | .63 | .58 | .56 | .66 | .51 | .46 | .52 |  | .67 | .84 | .61 |  | .42 | .38 | .43 |
| 4. Coherence | .68 | .83 | .73 |  | .62 | .62 | .69 | .65 | .60 | .58 |  | .75 | .82 | .70 |  | .38 | .40 | .42 |
| 5. Inappropriate | .54 | .65 | .62 | .68 |  | .63 | .72 | .59 | .60 | .67 |  | .89 | .59 | .74 |  | .28 | .26 | .29 |
| 6. Stereotyped | .59 | .76 | .62 | .76 | .69 |  | .68 | .61 | .55 | .66 |  | .73 | .66 | .86 |  | .42 | .36 | .40 |
| 7. Use of language | .63 | .74 | .67 | .77 | .71 | .76 |  | .64 | .63 | .63 |  | .91 | .68 | .76 |  | .38 | .38 | .41 |
| 8. Nonverbal | .54 | .66 | .62 | .72 | .70 | .75 | .81 |  | .71 | .65 |  | .83 | .60 | .77 |  | .31 | .30 | .33 |
| 9. Social | .46 | .60 | .50 | .66 | .65 | .68 | .71 | .77 |  | .56 |  | .73 | .52 | .82 |  | .26 | .24 | .26 |
| 10. Interests | .41 | .60 | .46 | .65 | .70 | .69 | .67 | .67 | .70 |  |  | .74 | .54 | .87 |  | .17^a^ | .17^a^ | .18^a^ |
| **CCC-2 Subscales** | | | | | | | | | | | | | | | | | | |
| 11. Pragmatic | .63 | .76 | .70 | .80 | .88 | .81 | .93 | .92 | .78 | .75 |  |  | .71 | .86 |  | .37 | .36 | .39 |
| 12. Language | .85 | .92 | .85 | .91 | .70 | .77 | .80 | .72 | .63 | .60 |  | .82 |  | .68 |  | .51 | .51 | .53 |
| 13. Autism | .55 | .73 | .59 | .77 | .76 | .88 | .80 | .82 | .90 | .89 |  | .88 | .75 |  |  | .33 | .30 | .33 |
| **Language Z scores** | | | | | | | | | | | | | | | | | | |
| 14. Vocabulary | .47 | .42 | .48 | .48 | .28 | .31 | .35 | .34 | .24 | .15^a^ |  | .36 | .52 | .26 |  |  | .74 | .90 |
| 15. Grammar | .42 | .37 | .45 | .45 | .21 | .28 | .27 | .27 | .18^a^ | .11^a^ |  | .27 | .48 | .21 |  | .68 |  | .89 |
| 16. Composite | .48 | .44 | .50 | .51 | .29 | .34 | .37 | .33 | .24 | .17^a^ |  | .36 | .54 | .28 |  | .87 | .87 |  |

a= p<.01, all other p<.001, bottom triangle = teacher reported CCC-2 sample, top triangle = parent reported CCC-2 sample

**Appendix S4**

We compared children in each quadrant of Figure 3 to determine if children with combined language and pragmatic deficits had more severe behaviour and academic outcomes relative to peers with relatively circumscribed deficits in either domain.

This analysis was repeated for Parent and Teacher samples and used a weighted approach, with the key comparisons being Quadrant A (language difficulties) and Quadrant B (pragmatic difficulties) when compared with Quadrant C (difficulties in both domains).

Early Years Profiles

Those in Quadrant A and B did not differ significantly on their Early Years Profiles (parent data: χ^2^(df=1)=2.89, p=.089; teacher: χ^2^(df=1)=0.01, p=.942), nor did Quadrant A and C (parent data: χ^2^(df=1)=0.00, p=1; teacher data: χ^2^(df=1)=0.55, p=.459).

SATS attainment

Those in Quadrant A and B did not differ significantly on their SATS attainment (parent data: χ^2^(df=1)=2.92, p=.089; teacher data: χ^2^(df=1)=0.47, p=.492, nor did Quadrant A and C (parent data: (χ^2^(df=1)=1.50, p=0.221; teacher data: χ^2^(df=1)=0.66, p=.417).

Strengths and difficulties Questionnaires

T-tests revealed participants in Quadrant C had significantly higher SDQ scores than those in Quadrant A (parent data: t(42.6) = 4.30, p<.001; teacher data: t(71.4) = 3.13, p=.003). The difference in scores between Quadrant B and C was not significant in the parent sample ( t(12.1)=0.05, p=.957), however in the teacher sample SDQ scores were actually significantly higher in Quadrant B (teacher data: t(11.7) = 2.87, p=.014).

This pattern of findings suggests that whether pragmatic skills are accompanied by structural language difficulties or not, there is a considerable impact on academic and behavioural outcomes. We interpret these results cautiously due to the small group sizes in each of the Quadrants.

**Appendix S5**

In our Functional Impact section we highlighted the group differences between children with low and typical pragmatic abilities. Here we go a step further to examine the unique contribution of pragmatic competence in three functional outcomes: SDQ total raw score (an index of problem behaviours); SATS attainment (a binary variable indicating if the child achieved all five expected attainments at the end of Year 2 and EYFS profile (a binary variable indicating whether the child achieved a ‘good level of development’ at the end of reception year).

For this analysis we used linear regression models with the following additional covariates: SES (as indicated by idaci rank) and non-verbal IQ. Two measures of structural language (expressive and receptive vocabulary; expressive and receptive grammar) were entered as predictors as well as the CCC-2 pragmatic composite. We did not include the CCC-2 autism composite in this analysis due to high collinearity with the pragmatic composite (r>.8 in both samples).

SDQ total raw score was log transformed due to being positively skewed. Binary outcomes were analysed with logistic regression models.

We completed separate analyses for the Teacher and Parent samples.

Results

Results are presented in Table S8. below. The significant predictors of SDQ total difficulties were pragmatics and SES in the Parent sample and pragmatics and grammar skills in the Teacher sample. Both models explained approximately 25% of the variance in scores.

Conversely pragmatics was not a significant predictor of SATS attainment, in either the Parent or the Teacher sample. Grammar was the only predictor of SATs success in the Teacher sample (pseudo r^2^ = .43) and parent sample (pseudo r^2^ = .30).

Finally, a good level of development on the EYFS screening was predicted by grammar and pragmatics skills in both the Parent and the Teacher sample, with pseudo r^2^ of .36 and .38 respectively.


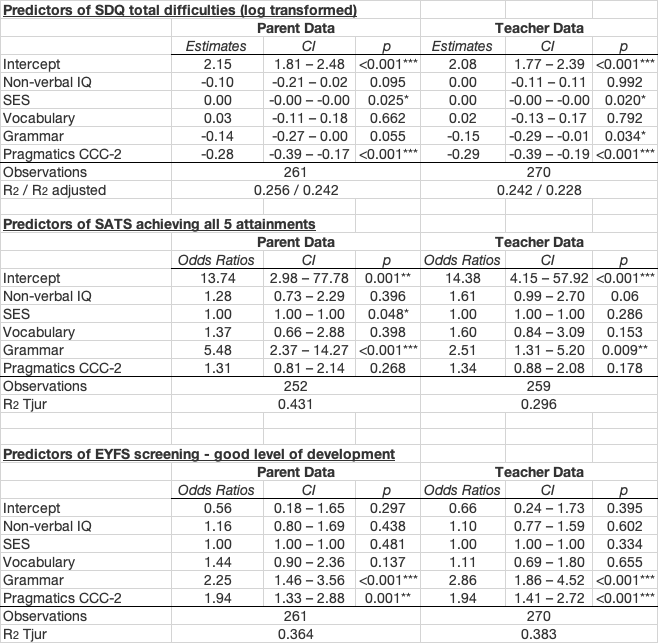
 **Table S8.** Linear regresson models predicting SDQ total difficulties scores, EYFS screening outcomes and achieving all five SATS attainments

Discussion

The findings above demonstrate the importance of pragmatic skills in conjunction with other linguistic and cognitive factors in early academic and social development.

1. An additional covariance between Inappropriate language and interests was required in order make the model converge with Teacher data. [↑](#footnote-ref-1)
